# X-ray and Hydrogen-bonding Properties of 1-((*1H*-benzotriazol-1-yl)methyl)naphthalen-2-ol

**DOI:** 10.3390/molecules14031234

**Published:** 2009-03-23

**Authors:** Augusto Rivera, Yorley Duarte, Diego González-Salas, Jaime Ríos-Motta, Guillermo Zaragoza

**Affiliations:** 1 Departamento de Química, Universidad Nacional de Colombia, Ciudad Universitaria, Carrera 30 No. 45-03, Bogotá D.C., Colombia; E-mails: yaduartea@unal.edu.co (D.Y.), dagonzalezsa@unal.edu.co (G-S.D.) jariosmo@unal.edu.co (R-M.J.); 2 Unidade de Difracción de Raios X, Edificio CACTUS, Universidade de Santiago de Compostela, Campus Sur, Santiago de Compostela, Galicia, E-15782, Spain; E-mail: g.zaragoza@usc.es (G.Z.)

**Keywords:** Mannich bases, Benzotriazole, Intra-intermolecular hydrogen bond, 1-((*1H*-benzotriazol-1-yl)methyl)naphthalen-2-ol, Single crystal X-ray diffraction.

## Abstract

The solid state structure of 1-((*1H*-benzotriazol-1-yl)methyl)naphthalen-2-ol, C_17_H_13_N_3_O, shows that this Mannich base crystallizes forming intermolecular N⋯HO hydrogen bonds, rather than intramolecular ones. Factors contributing to this choice of hydrogen-bonding mode are discussed. The compound crystallizes in the monoclinic system, *P2_1_/c* space group, with lattice constants: *a* = 11.7934(9) Å, *b* = 14.3002(14) Å, *c* = 8.4444(8) Å, *β* = 106.243(5) deg, *V* = 1367.3(2) Å^3^, *Z* = 4, *F*(000) = 576, *R_1_* = 6.96%, *wR_2_* = 11.4%.

## Introduction

In recent years, much attention has been focused on the use of benzotriazole (**1**) methodology as a versatile synthetic tool. Benzotriazole-mediated amino-alkylations have greatly broadened the utility of Mannich-type condensations to the general *o*-aminoalkylation of phenols. Phenols are selectively aminoalkylated in the *ortho* position by the displacement of the benzotriazole moiety from *N*-[α-(dialkylamino)alkyl]-benzotriazoles with phenolate anions [1,2,3,4]. At this respect, an important application of the benzotriazole methodology is the use of *o*-(α-benzotriazolylalkyl)phenols (**2**) as a versatile intermediates for the preparation of 1,1-bis(2-hydroxyaryl)alkanes [5], *o*-substituted phenols [6] and *o*-quinone methides (*o*-QMs) [7].


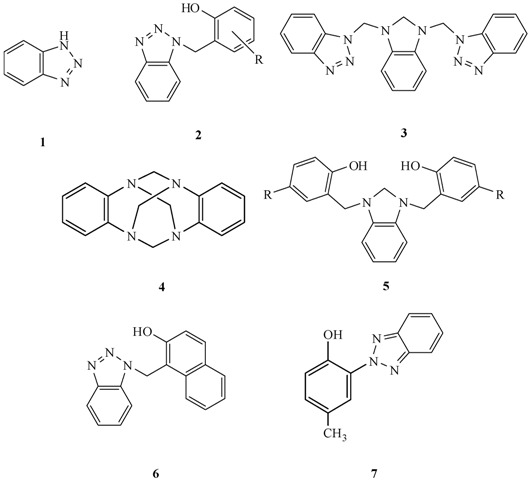


As part of a program aimed at the synthesis of salans in our laboratory, we envisioned that 1,3-bis((1*H*-benzotriazol-1-yl)methyl)-2,3-dihydro-1*H*-benzo[*d*]imidazole (**3**), prepared by condensation of 6H,13H-5:12,7:14-dimethanedibenzo-[*d,i*] [1,3,6,8]-tetraazecine (DMDBTA, **4**) and benzotriazole in dioxane [8], could serve as a versatile precursor for a new class of benzimidazolidine-based salans, such as **5**. Surprisingly, despite the fact that benzotriazole methodology provides a convenient synthesis of phenolic Mannich bases, our attempts to prepare the phenolic Mannich bases such as **5** using the *N*-benzotriazolylmethyl derivative **3** and several phenols were unsuccessful. In fact, benzotriazole did not act in its usual role as a leaving group [9]. However, to our pleasant surprise, the corresponding Mannich product (**6**) was obtained in good yield when 2-naphthol was used instead phenols.

On the other hand, compound **6** contains hydrogen bonding donor/acceptor sites in the molecule which leads to inter- or intramolecular hydrogen-bond interactions. In order to gain additional information about both the capability of BTZs to act as hydrogen-bond acceptors, and the structural consequences of the hydrogen bonding on these Mannich-type bases, we were able to obtain crystals of 1-((*1H*-benzotriazol-1-yl)methyl)naphthalen-2-ol (**6**) and here report an X-ray structural analysis of **6** and its crystallographic characterization.

## Results and Discussion

Inspired by the synthetic utility of benzotriazole, we began to explore reactions of aminal compounds with benzotriazole in solution, leading to new synthetic applications and perspectives for these compounds.

**Scheme 1:**
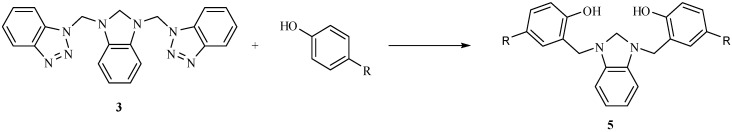
Planned synthetic route to Mannich-type bases.

However, when we carried out the reaction between **3 **and some electron-rich and electron-deficient phenols at reflux in isopropanol, according to known methodology [10,11], neither Mannich bases (**5**) nor any other compound produced by the aminomethylation of phenols were obtained. However, 2-naphthol was an exception, yielding crystalline flakes (mp 215-217 °C). IR and ^1^H-NMR spectral analysis indicated that product, compound **6**, was identical to that obtained by a three component reaction of **1** with formaldehyde and 2-naphthol [12].

Our interest in understanding the role of the O–H⋯N intra- and intermolecular interactions in the course of Mannich-type reactions led us to continue. The intramolecular hydrogen bond has been described, in Mannich bases, to have certain structural and mechanistic implications, such as an increase in stability. For 1-((*1H*-benzotriazol-1-yl)methyl)naphthalen-2-ol (**6**), the structure can be described in terms of the presence or absence of an intra- or intermolecular hydrogen bond. Preferred hydrogen-bond modes, which play a role in chemical reactions as the active site for initiating reactions, were derived from crystal structures and may be useful for determining the preferred modes of association of the individual functional groups in complex molecules [13]. The benzotriazolyl ring has a high possibility of forming an intramolecular hydrogen bond; in fact, earlier studies have demonstrated that some *1H*- and *2H*-benzotriazolyl derivatives exhibit these interactions [14,15,16,17]. Previous theoretical and experimental studies have demonstrated that the proton affinity of 1*H*-benzotriazole is larger than 2*H*-benzotriazole by about 10 kCal/mol [18]. Thus, we expected that the hydrogen atom of the hydroxyl group of 2-naphthol could be involved in a hydrogen-bond with the neighboring benzotriazole ring nitrogen atoms or that it may interact with another molecule in its proximity, thus creating an intermolecular hydrogen bond.

**Figure 1:**
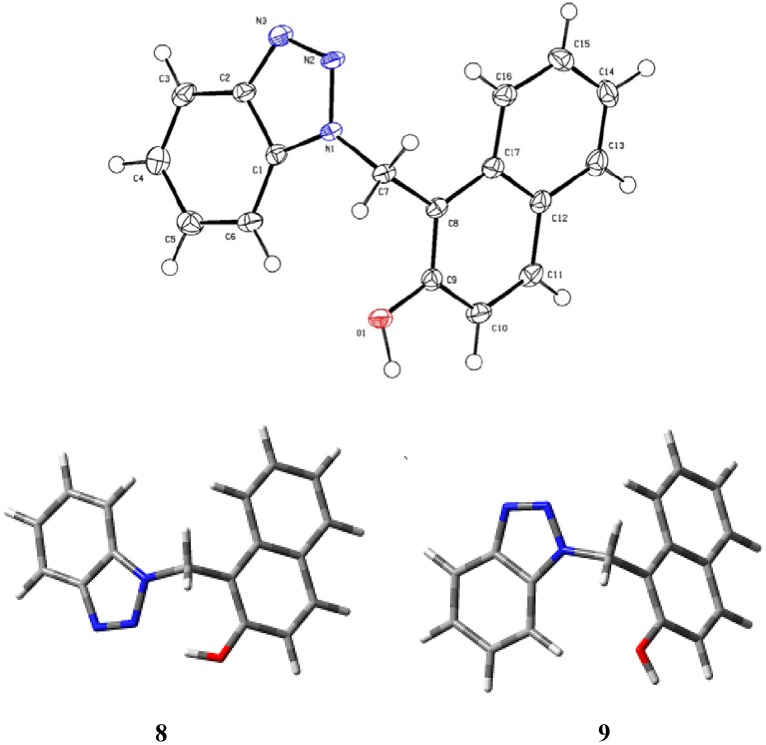
Molecular structure of **6** at 100 K. Thermal ellipsoids are shown at 50% probability.

Single crystal X-ray diffraction analysis reveals the absence of intramolecular hydrogen bonding in the solid state, as shown in Figure 1. There are several possible causes for this phenomenon *e.g*, the formation of an intramolecular hydrogen bond tends to decrease the aromaticity of the benzotriazol ring. In order to evaluate this hypothesis, we used a computational quantum mechanical approach to examine the stability of the hydrogen-bonded and non-hydrogen-bonded structures of the title compound. The structures were energy minimized by using a hybrid density functional method at the B3LYP/6-31G (d,p) level of theory. The gas-phase calculations suggest that the hydrogen bonded structure **8** is more stable that the non-hydrogen-bonded structure **9** (ΔE 2.44 kcal/mol). Thus this hypothesis is not a valid explanation for the results of X-ray diffraction experiments.

**Figure 2:**
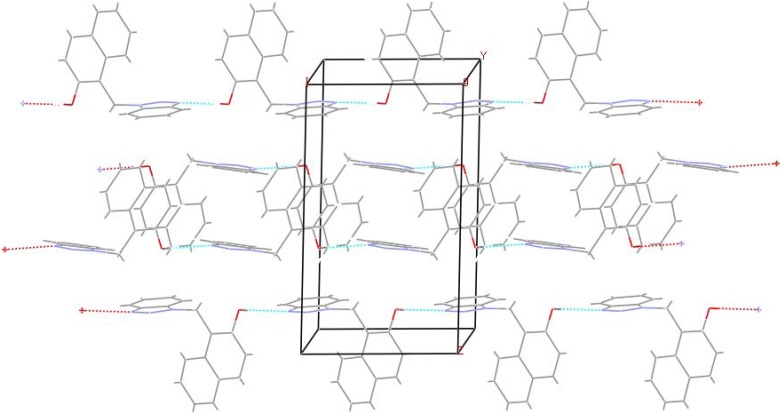
Crystal packing diagram of **6**, showing an extended hydrogen-bonded network.

Thus other structural features most associated with molecular packing stability should be invoked. For example, an intermolecular interaction helps to organize the molecules, forming a well-defined crystal packing. It is known that intermolecular hydrogen bonds play an important role in the construction of ordered organic networks [19]. A search of the packing diagram of **6 **(Figure 2), using the Mercury program [20], shows that the crystal lattice forms an infinite hydrogen-bonded network, where hydrogen bonding interactions between nitrogen atoms and hydroxyl groups led to the formation of a chain extend along the crystal b-axis. Thus, the orientation of the hydroxyl-substituted ring is fixed by the intermolecular hydrogen bond to ***N3***. These intermolecular OH⋯N hydrogen bonds influence the conformation of the molecule and connect molecules in a *zig-zag* chain parallel to the ab plane. In the absence of intramolecular hydrogen bonding, the aromatic cores are better accommodated in the gauche conformation to avoid a strong repulsion between rings. This conformation is evident by inspection of torsion angle between the two aromatic rings (C1-N1-C7-C8, 71.8(2)°). The observed C-O bond in **6** (1.365(2) Å) is considerably shorter than C-O bond in phenol (1.381 Å) [21]. This bond, however, is slightly longer than the C-O bond in 2-((*2H*-benzotriazol-2-yl)methyl)-4-methylphenol (**7**) (C-O 1.359 Å) [17]. A comparison of the structural parameters of the intra- and inter hydrogen-bonded interactions in **6** and **7**, respectively (Table 2), shows that the nearly linear hydrogen bond in **6** is slightly weaker that in **7**, where the hydrogen bond is substantially non-linear [22].

**Table 1:** Structural characteristics of **6**.

*Bond lengths (Å)*		*Bond angles (deg)*	
O(1)-C(9)	1.365(2)	N(2)-N(1)-C(7)	120.64(13)
N(1)-N(2)	1.348(2)	N(2)-N(1)-C(1)	110.69(14)
N(1)-C(7)	1.471(2)	C(1)-N(1)-C(7)	128.67(14)
N(1)-C(1)	1.363(2)	N(3)-N(2)-N(1)	108.36(13)
N(3)-N(2)	1.312(2)	N(2)-N(3)-C(2)	108.76(14)
N(3)-C(2)	1.377(2)	C(9)-C(8)-C(17)	119.23(15)
C(8)-C(9)	1.384(2)	C(9)-C(8)-C(7)	119.82(15)
C(8)-C(17)	1.429(2)	C(17)-C(8)-C(7)	120.93(15)
C(8)-C(7)	1.505(2)	O(1)-C(9)-C(8)	118.94(15)
C(9)-C(10)	1.411(2)	O(1)-C(9)-C(10)	119.94(15)
C(10)-C(11)	1.362(2)	C(8)-C(9)-C(10)	121.13(16)
C(11)-C(12)	1.418(2)	C(11)-C(10)-C(9)	120.20(16)
C(12)-C(13)	1.416(2)	C(10)-C(11)-C(12)	120.96(17)
C(12)-C(17)	1.423(2)	C(13)-C(12)-C(11)	121.26(16)
C(13)-C(14)	1.365(3)	C(11)-C(12)-C(17)	119.11(16)
C(14)-C(15)	1.403(3)	C(13)-C(12)-C(17)	119.61(16)
C(15)-C(16)	1.372(3)	C(14)-C(13)-C(12)	120.68(18)
C(16)-C(17)	1.420(2)	C(13)-C(14)-C(15)	120.20(17)
C(1)-C(6)	1.401(2)	C(16)-C(15)-C(14)	120.53(17)
C(1)-C(2)	1.397(2)	C(15)-C(16)-C(17)	121.02(17)
C(5)-C(6)	1.372(2)	C(16)-C(17)-C(12)	117.89(16)
C(4)-C(5)	1.413(3)	C(16)-C(17)-C(8)	122.84(16)
C(3)-C(4)	1.372(3)	C(12)-C(17)-C(8)	119.26(15)
C(2)-C(3)	1.404(2)	N(1)-C(7)-C(8)	112.34(13)
		N(1)-C(1)-C(2)	104.29(15)
		N(1)-C(1)-C(6)	132.97(16)
		C(2)-C(1)-C(6)	122.68(16)

**Table 2:** Structural parameter of the hydrogen-bonded interactions in **6** and **7**.

Structure	d(Å)			Angle
	O-H	H⋯N	O⋯N	N⋯HO
**6^a^**	0.99(2)	1.77(2)	2.7618(19)	176(2)
**7^b^**	0.85(3)	1.85(4)		149(3)

^a^ present work; ^b^ Ref [17]

Due to the fact that the pKa value of benzotriazole in aqueous solution (pK = 0.06 [23]) is lower than that of aryl amines (pKa aniline = 4.60 [24]), and according to our X-ray results, we expected that the preferred site of hydrogen bonding in 1,3-bis((1*H*-benzotriazol-1-yl)methyl)-2,3-dihydro-1*H*-benzo[*d*]imidazole (**3**) would be the aryl nitrogen, which is a stronger H-bond acceptor than the nitrogen atoms of the benzotriazole ring. 

**Scheme 2:**
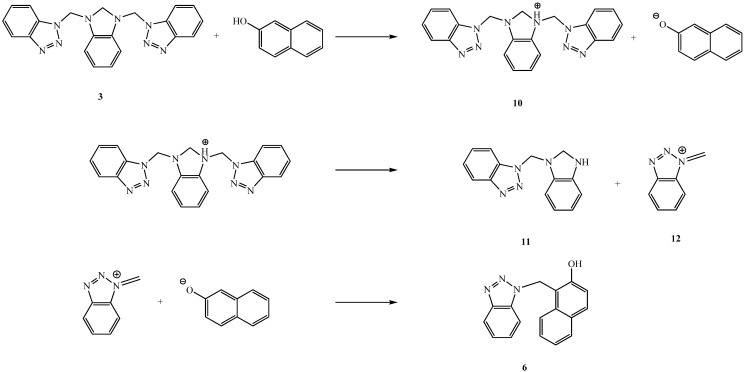
Proposed mechanism for the formation of **6**.

In view of the last assumptions, the Mannich reaction between **3** and 2-naphthol should occur via heterolytic CH_2_―Btz bond dissociation with substitution of the benzotriazole groups by naphthol, but the isolated product does not correspond to this sequence and our observation is thus not consistent with a concerted mechanism wherein the aminomethylation of 2-naphthol is mediated by hydrogen bond formation with an NCH_2_Btz fragment, which has been proposed to explain the high regioselectivity of Mannich reactions [25]. Thus to explain our experimental observations, a reaction mechanism for the formation of **6** is proposed in Scheme 2, in which 2-naphthol induces a proton transfer followed by a fast cleavage of **3** to benzimidazole compounds, such as 1-((2,3-dihydro-1*H*-benzo[*d*]imidazol-1-yl)methyl)-1*H*-benzo[*d*][1,2,3]triazole (**11**). 

Unfortunately, the isolation and exact chemical nature of benzimidazole intermediate has not yet been established. In contrast, the main products formed from the reaction of ion **12** are the result of simple nucleophilic attack by the naphtholate anion to afford **6**. Finally, although systems of the type Bt-CH_2_-N react with a wide variety of nucleophiles, giving replacement of the benzotriazolyl group [3], the results indicate that in this reaction the benzotriazole methodology cannot be considered as a versatile synthetic tool.

## Experimental

### General

Melting points were determined with an Electrothermal apparatus and are uncorrected. 1,3-Bis((1*H*-benzotriazol-1-yl)methyl)-2,3-dihydro-1*H*-benzo[*d*]imidazole (**3**) was prepared following the procedure described in literature [8]. 2-Naphthol was purchased from Merck and used without further purification. 

### Synthesis

Following the methodology of the Katritzky group [10,11] a solution of 2*-*naphthol (300 mg, 2 mmol) in isopropanol (5 mL) was added to a stirred solution of **3** (412 mg, 1 mmol) in isopropanol (5 mL), followed by addition of a few drops of triethylamine to ensure a basic medium. The resulting mixture was heated under reflux during 12 h. After cooling to the room temperature, the obtained solid (170 mg, 62%) was filtered off and washed with cold isopropanol. Recrystallization from the same solvent gave a white crystal flakes, mp 215-217 °C.

### X-ray crystallography

Suitable crystals of **6** were obtained by slow crystallization from isopropanol. Data for 6 was collected on a Bruker APPEX CCD Difractometer at 100K, using graphite-monochromate Mo-Kα radiation (k = 0.71073 Å) from a fine-focus sealed tube source. The computing data and reduction was made by APPEX2 [26] software, an a empirical absortion correction was applied using SADABS. [27] The structure was solved by SIR97 [28] an finally was refined by full-matrix, least-squares based on F^2^ by SHELXL. [29] All non hydrogen atoms were anisotropically refined and the hydrogen atoms positions were included in the model by electronic density or were geometrically calculed and refined using a riding model.. Crystallographic data (excluding structure factors) have been deposited at the Cambridge Crystallographic Data Centre. The CCDC deposition number is 723156. Copies of the data can be obtained free of charge on application to the CCDC, 12 Union Road, Cambridge CB2 IEZ, UK. Fax: +44-(0)1223-336033 or e-mail: deposit@ccdc.cam.ac.uk. Crystal data and details concerning data collection and structure refinement are given in Table 3, and bond distances and angles are listed in Table 1.

**Table 3:** Crystal data and structure refinement for **6**.

Empirical formula	C_17_H_13_N_3_O
Temperature	100(2) K
Formula weight	275.30
Wavelength	0.7107 Å
Crystal system	Monoclinic
Space group	*P2_1_/c*
Unit cell dimensions	*a* = 11.7934(9) Å
	*b* = 14.3002(14) Å
	*c* = 8.4444(8) Å
	β = 106.243(5)°
Volume	1367.3(2) Å^3^
*Z*	4
Calculated density	1.337 g/cm^3^
Absorption coefficient	0.09 mm^-1^
*F*(000)	576
Crystal size	0.36 x 0.12 x 0.07 mm
*θ* range for data collection	2.3 - 23.9^o^
Limiting indexes	0 ≤ h ≤ 14, -17 ≤ k ≤ 0, -10 ≤ l ≤ 10
Reflections collected/unique	9100/2696 [R(int) = 0.0634]
Completeness to *θ* = 30.50	100%
Final shift / error, max and avg	0.002, 0.000
Refinement method	Full-matrix least-squares on *F^2^*
Data / restraints / parameters	2696/0/194
Goodness of fit on *F^2^*	1.038
Final *R* indices [I >2sigma (I)]	*R1* =4.61%, *wR2* = 10.58%
R indices (all data)	*R1* = 6.96%, *wR2* = 11.41%
Largest peak and hole	0.30 and -0.31 e^-^Å^-3^

### Theoretical calculations

For the theoretical calculations, geometries of the hydrogen bonded (**8**) and non-hydrogen bonded (**9**) structures were optimized at the B3LYP level with the 6-31G(d,p) basis set using Gaussian-03 software [30].
